# Inhibition of the protective effects of preconditioning in ischemia–reperfusion injury by chronic methadone: the role of pAkt and pSTAT3

**DOI:** 10.1038/s41598-024-65349-x

**Published:** 2024-06-21

**Authors:** Mahboobeh Yeganeh-Hajahmadi, Zeinab Kordestani, Yasmin Moosavi-Saeed, Farzaneh Rostamzadeh

**Affiliations:** 1 Physiology Research Center, Institute of Neuropharmacology, Kerman University of Medical Sciences, Jehad Blvd, Ebn Sina Avenue, Kerman, 76137-53767 Iran; 2 Cardiovascular Research Center, Institute of Basic and Clinical Physiology Sciences, Kerman University of Medical Sciences, Kerman, Iran; 3 Endocrinology and Metabolism Research Center, Institute of Basic and Clinical Physiology Sciences, Kerman University of Medical Sciences, Kerman, Iran

**Keywords:** Methadone, Ischemia–reperfusion injury, Ischemic preconditioning, Isolated heart, RISK (reperfusion injury salvage kinase), SAFE (survivor activating factor enhancement), Physiology, Cardiology

## Abstract

Cardiac ischemic preconditioning (Pre) reduces cardiac ischemia–reperfusion injury (IRI) by stimulating opioid receptors. Chronic use of opioids can alter the signaling pathways. We investigated the effects of chronic methadone use on IRI and Pre. The experiments were performed on isolated hearts of male Wistar rats in four groups: IRI, Methadone + IRI (M-IRI), Pre + IRI (Pre-IRI), Methadone + Pre + IRI (M-Pre-IRI). The infarct size (IS) in the Pre-IRI group was smaller than the IRI group (26.8% vs. 47.8%, *P* < 0.05). In the M-IRI and M-Pre-IRI groups, the infarct size was similar to the IRI group. Akt (Ak strain transforming) phosphorylation in the Pre-IRI, M-IRI, and M-Pre-IRI groups was significantly higher than in the IRI group (0.56 ± 0.15, 0.63 ± 0.20, and 0.93 ± 0.18 vs 0.28 ± 0.17 respectively). STAT3 (signal transducer and activator of transcription 3) phosphorylation in the Pre-IRI and M-Pre-IRI groups (1.38 ± 0.14 and 1.46 ± 0.33) was significantly higher than the IRI and M-IRI groups (0.99 ± 0.1 and 0.98 ± 0.2). Thus, chronic use of methadone not only has no protective effect against IRI but also destroys the protective effects of ischemic preconditioning. This may be due to the hyperactivation of Akt and changes in signaling pathways.

## Introduction

Ischemic heart diseases are the number one threat to human health worldwide^1^. Preservation of ischemic heart muscle cells and limiting the size of the infarcted area depends on rapid and effective reperfusion^2^. However, reperfusion is inherently risky, as it can lead to further damage to the heart muscle, called ischemia–reperfusion injury (IRI)^3,4^.

One way to reduce IRI is ischemic preconditioning (Pre)^5^. For the first time, Murry et al*.* demonstrated the beneficial effect of Pre on IRI. Murray's research illustrated that a brief period of coronary artery blockage lasting 4–5 min, followed by 5 min of reperfusion, can yield positive results^6^. Pre not only reduces the size of the infarcted area but also improves coronary microcirculation^7^. It is believed that Pre induces a series of intracellular signaling cascades, resulting in a memory effect that ultimately reduces IRI^8^. Receptor-based and non-receptor-dependent factors that play a role in ischaemic conditioning include opioids, adenosine, bradykinin, acetylcholine, TNF cytokines, and various other factors. Additionally, mechanical stretch, ROS, reactive nitrogen species, and extracellular Ca^2+^ can initiate ischaemic conditioning signaling without the need for a receptor^9^. They transmit signals through cytosolic proteins, mainly kinases, to various intracellular targets like mitochondria, sarcoplasmic reticulum, and the nucleus. The primary signal cascades involved are the protein kinase C (PKC)–endothelial NO synthase (eNOS)–protein kinase G (PKG) pathway, the reperfusion injury salvage kinase (RISK) pathway, and the survival activating factor enhancement (SAFE) pathway^9^. The RISK and SAFE pathways are two main signaling pathways that activated in parallel and are involved in Pre-induced cardiac protection: Akt and STAT3 are the main mediators of these pathways respectively^9,10^.

Although the beneficial effects of preconditioning have been identified, unfortunately, translation of ischemic conditioning interventions and drugs targeting ischemic conditioning signaling to benefit patients with acute myocardial infarction have been generally unsatisfactory^11^. Therefore, understanding signaling pathways and confounding factors is of great importance.

Large amounts of endogenous opioids and their receptors are expressed in the heart, and laboratory studies on several species have shown that this system’s activity is one of the most important systems for protecting the heart in preconditioning^12–15^. Opioid receptors are regarded as a crucial therapeutic target for cardioprotection against ischemia. The use of receptor antagonists has been shown to hinder the beneficial effects of ischemic preconditioning^16^. Furthermore, administration of morphine and methadone 5 min before ischemia has resulted in a reduction in the size of the infarcted area following ischemia–reperfusion injury^17^.

When opioids are used acutely, they decrease cyclic adenosine monophosphate (cAMP) levels. In addition, they lead to hyperpolarization of cells through the activity of potassium and calcium channels^18^. However, chronic stimulation of opioid receptors by morphine and other opioid ligands leads to adaptive changes in opioid receptors, which ultimately leads to decreased response. These changes are compensatory mechanisms to control the activity of the receptor, protecting the receptors against excessive stimulation, promoting signal termination, and regulating their expression^19^. Therefore, with chronic use of opioids, mechanisms such as desensitization, internalization, re-sensitization, or downregulation of opioid receptors are initiated. These mechanisms lead to a decrease in the pharmacological activity of morphine and other opioid drugs^19^. In addition, chronic use of opioids can be associated with other changes. For example, it has been shown that chronic use of morphine (5 days) in rats can increase the heterodimerization of mu and delta receptors in the central nervous system^20^. It has been shown that heterodimerization of receptors can change the signaling pathway^21,22^.

According to the World Health Organization report, in 2021, around sixty million people in the world used opioids^23^, and opioid use disorder (OUD) is one of the health challenges in the world^24^. Methadone is a synthetic opioid that was developed in the 1940s as an analgesic^25^, and currently, it is used to treat chronic pain and OUD (detoxification and maintenance treatment)^24^. Numerous studies on humans and laboratory animals have shown that chronic use of opioids is an independent risk factor for developing cardiovascular diseases^26–29^. Considering the high consumption of methadone, the association of chronic use of opioids with changes in the endogenous opioid system, and the relatively higher prevalence of cardiovascular problems in opioid users^26–29^, chronic methadone use may modify the effects of ischemic preconditioning. Therefore, this study was conducted to investigate the effects of chronic methadone use on ischemia–reperfusion injury and the protective effects of ischemic preconditioning on ischemia–reperfusion injury in the heart of male rats. For this purpose, the size of the infarcted area, functional indicators of the heart, heart muscle damage (cardiac troponin I level), and the ratios of phosphorylated Akt and STAT3 to total Akt and STAT3, which are known as indicators of RISK and SAFE pathway activation, respectively, were investigated.

## Methods and materials

### Animals

Forty-eight 8-week-old male Wistar rats (Rattus norvegicus) weighing between 180 and 200 g were purchased from the animal house of Kerman University of Medical Sciences. The animals were maintained under 12-h light/dark cycles and had free access to food and water. All experiments were performed following the national guidelines for animal studies and the study was approved by the Ethics Committee of Kerman University of Medical Sciences (ethical code: IR.KMU.REC.1399.063) and adhered to the ARRIVE guidelines. The guidelines were carefully followed to ensure transparency, reproducibility, and ethical standards in our research process and reporting. After a week of adaptation, the animals were randomly placed in four groups of twelve (7 hearts for measuring functional heart indices and chemical and molecular investigations and 5 hearts for staining and assessment of the size of the infarcted area) and they received drinking water or drinking water containing methadone for four weeks. At the age of thirteen weeks, experiments were performed on the isolated hearts.

1. Ischemia–reperfusion injury (IRI) group: after receiving 4 weeks of normal water as methadone solvent, the rats’ hearts were transferred to the Langendorff system and exposed to ischemia–reperfusion.
2. Methadone + IRI (M-IRI) group: After receiving 4 weeks of increasing doses of methadone, the rats’ hearts were transferred to the Langendorff system and exposed to ischemia–reperfusion.
3. Pre + IRI (Pre-IRI) group: After receiving 4 weeks of water as methadone solvent, the rats' hearts were transferred to the Langendorff system, and before long-term global ischemia–reperfusion induction, the hearts were exposed to global ischemia for three periods of five minutes, all of the ischemia periods were followed by five minutes of reperfusion.
4. Methadone + Pre + IRI (M-Pre-IRI) group: In this group, the animals were exposed to incremental doses of methadone for four weeks, similar to the second group, and at the end of the fourth week, the hearts of these animals were exposed to ischemic preconditioning and ischemia–reperfusion as in the third group.

**Figure 1:**
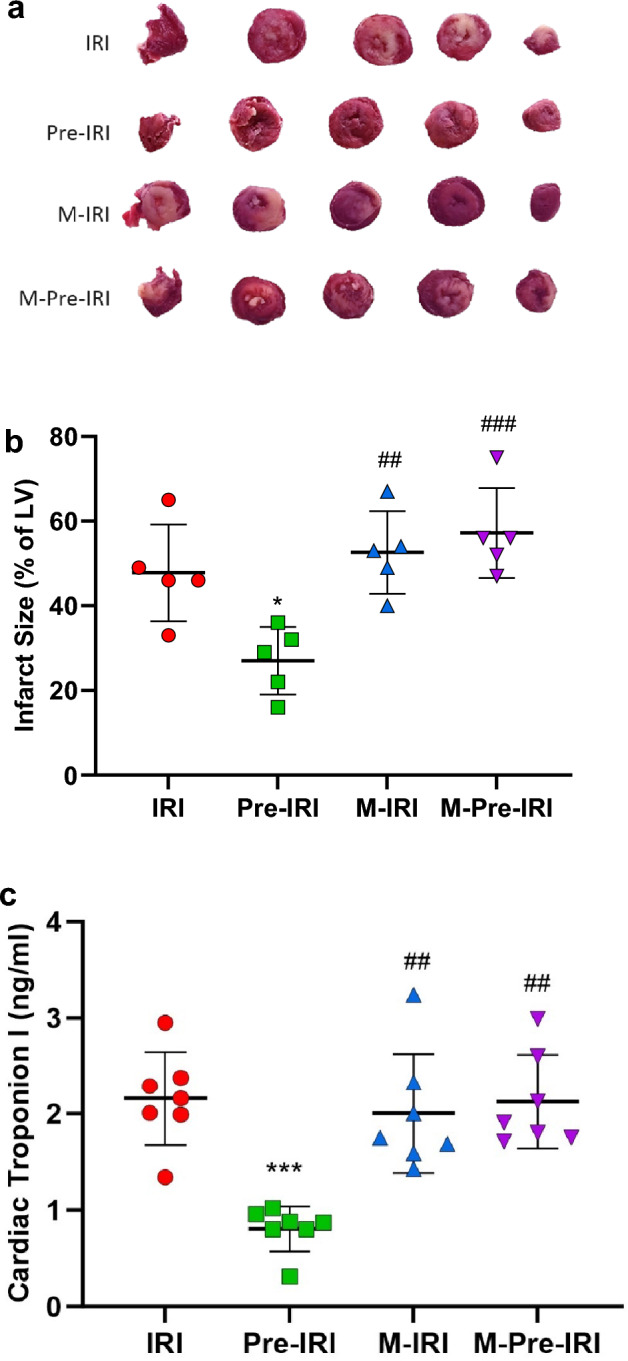
Effects of 4 weeks methadone consumption on ischemia reperfusion-induced cardiac damage. (**a**) TTC staining shows infarct size in different groups. The heart transverse sections in one animal of each group. (**b**) Quantification of infarct size. (**c**) cardiac troponin I in the studied groups. *IRI* Ischemia–reperfusion injury, *Pre-IRI* Ischemic preconditioning + ischemia–reperfusion injury, *M-IRI* Methadone + ischemia–reperfusion injury, *M-Pre-IRI* Methadone + ischemic preconditioning + ischemia–reperfusion injury *P < 0.05, ***P < 0.001 vs. IR, ^##^*P* < 0.01, ^###^*P* < 0.001 *vs*. Pre-IR, *n* = 5 in each group for infarct size and *n* = 7 for troponin assessments.

### Chronic use of methadone

Methadone was added to the animals' drinking water incrementally. On the first two days 0.1 mg/ml, on days three and four 0.2 mg/ml, on days five and six 0.3 mg/ml, and from day seven to the end of the study 0.4 mg/ml of methadone was added to the drinking water^30^.

### Inducing ischemia–reperfusion injury and preconditioning

Ischemia–reperfusion injury and preconditioning were induced on the isolated heart in the Langendorff system. To prevent blood clots in the animal, first, 500 units of heparin per 100 g of weight were injected intraperitoneally. Ten minutes after the heparin injection, the rats were anesthetized using sodium thiopental at the dose of 50 mg per kilogram of body weight^31^. Then, by making a slit in the abdomen that extended to both sides of the chest, the rat's heart was quickly removed and transferred to the Langendorff system. The heart was retrogradely perfused with Krebs solution (pH 7.4) at a temperature of 37 °C (containing glucose 11, NaCl 118 mmol/L, KCL 4.8 mmol/L, MgSO_4_ 1.2 mmol/L, KH_2_PO_4_ 1.2 mmol/L, NaHCO_3_ 25 mmol/L, and CaCl_2_ 1.2 mmol/L), which was aerated with carbogen gas mixture (95% oxygen and 5% carbon dioxide), through the aorta. The perfusion pressure was kept constant at 70 mmHg during the experiment.

Left ventricular function was monitored through a ballon, which was connected to a pressure transducer and sent into the left ventricle through the left atrium. Before starting the experiments, the hearts were given 20 min to adapt to the conditions (stabilization phase). Then, to induce ischemia–reperfusion, the hearts were exposed to ischemia for 30 min, followed by reperfusion for 60 min. To create global ischemia, the Krebs solution flow into the aorta was cut off for 30 min and re-established to create reperfusion. In the preconditioning groups, immediately before the start of ischemia, the hearts were exposed to three periods of short-term ischemia–reperfusion (five minutes of ischemia, five minutes of reperfusion)^14^. During all the mentioned stages, the rate of left ventricular developed pressure (LVDevP), left ventricular end-diastolic pressure (LVEDP), and + dP/dtmax was recorded using the Power lab physiograph system.

### Heart damage determination

The degree of heart damage was assessed by measuring the level of cardiac troponin I in the coronary effluent at the end of the experiment. Triphenyl tetrazolium chloride (TTC) staining was used to measure the infarcted area of the heart. At the end of the experiments, the heart was removed from the device and 3-mm slices were prepared on the transverse axis. The slices were incubated for 15 min in 1.5% TTC solution in phosphate buffer at 37 °C. Then, the stained sections were placed in 4% paraformaldehyde for 48 h. Finally, using ImageJ software (https://imagej.net/ij/download.html), the surface area of the infarcted area was determined and expressed as a percentage of the total area of the heart. The cardiac troponin I level was measured using a cardiac troponin I ELISA kit (ab24652) manufactured by Abcam (United States).

### Measurement of Akt and STAT3 phosphorylation using the western blot method

Heart tissue samples were homogenized on ice in a cold RIPA buffer. The homogenate was centrifuged at 4 °C for 20 min at 14,000×*g*. The supernatant solution was separated, and its protein concentration was determined using the BCA Protein Quantification kit and according to the manufacturer’s instructions. An equal volume of 2 × Laemmli sample buffer was added to the lysate (containing 15 μg of protein) and boiled for 5 min.

The samples were resolved electrophoretically on a 12.5% SDS-PAGE gel and transferred to PVDF membranes (Cat. No.: 162-017777; Bio-Rad Laboratories, CA, USA). The membranes were then blocked with 5% BSA (Cat. No.: A-7888; Sigma Aldrich, MO, USA) in 0.1% Tween 20 for 1 h. Then, the membranes were incubated with Anti-AKT (Cat. No.: ab8805, Abcam), anti-p-AKT (Cat. No.: ab38449, Abcam), anti-STAT3 (Cat. No.: ab68153, Abcam), anti-p-SATAT3 (Cat. No.: ab76315, Abcam), and anti-beta actin-loading control antibodies (Cat. No.: ab8227, Abcam) for 1 h at room temperature. Subsequently, the membranes were washed three times with TBST, and incubated with goat anti-rabbit IgG H&L (HRP) (Cat. No.: ab6721; Abcam) secondary antibodies. The membranes were then incubated with enhanced chemiluminescence (ECL) for 1–2 min. Protein expression was normalized to β-actin. The density of protein bands was analyzed using Gel Analyzer version 2010a software (NIH, USA, https://www.softpedia.com/get/Science-CAD/GelAnalyzer.shtml#download); the percentage area under the curve of each band was divided by the percentage area under the curve of its corresponding actin band, and the calculated values were compared between groups^32^.

### Statistical analysis

Data were expressed as mean ± SD. All statistical analyses were done by SPSS 26 and GraphPad Prism10. After verifying the normal distribution of data using the Shapiro–Wilk test, one-way ANOVA was used to compare cardiac troponin I and infarct size. Hemodynamic data were analyzed using two-way repeated measure ANOVA. In the case of significance, pairwise comparisons were conducted by Tukey's post hoc test. *P*-values < 0.05 were considered to be statistically significant.

## Results

As expected, preconditioning significantly reduced the size of the infarcted area compared to ischemia–reperfusion (26.8% ± 8 vs. 47.8% ± 11.5, *P* < 0.05). Chronic use of methadone had no significant effect on the size of the infarcted area following IRI. However, the beneficial effects of preconditioning were lost. The size of the infarcted area in the M-IRI and M-Pre-IRI groups was 52.4% ± 9.8 and 57.4% ± 10.4 (*P* < 0.01 and *P* < 0.001 compared to Pre-IRI), respectively (Fig. 1a,b).

The cardiac troponin I levels in the cardiac effluent of the Pre-IRI group were significantly lower than the IRI group (0.81 ± 0.26 vs. 2.16 ± 0.53 ng/ml, *P* < 0.001). However, in the M-IRI and M-Pre-IRI groups (2.01 ± 0.68 and 2.13 ± 0.57 ng/ml, respectively) they were similar to the IRI group but significantly more than the Pre-IRI group (*P* < 0.01) (Fig. 1c).

**Figure 2:**
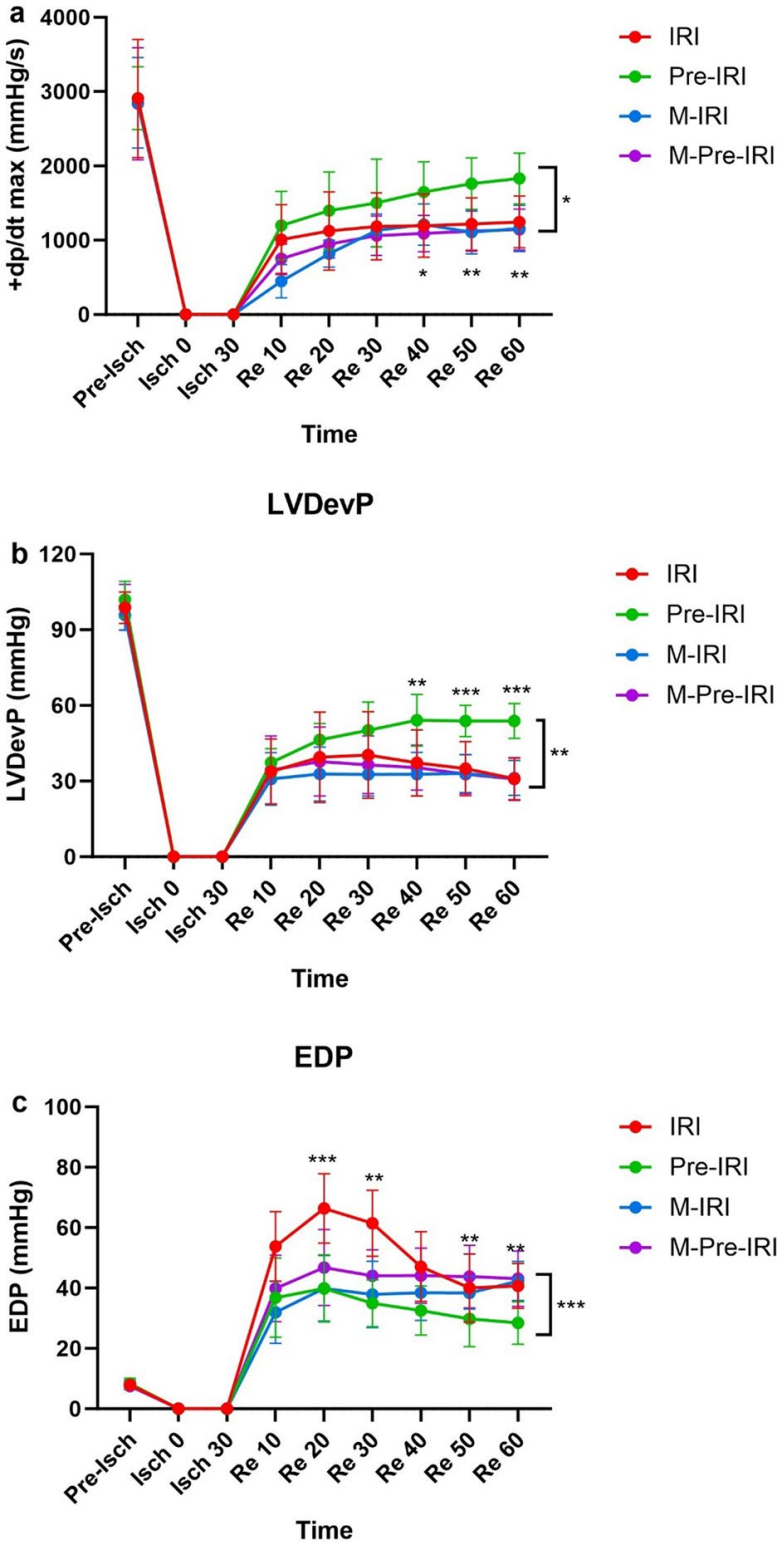
Effects of 4 weeks of methadone consumption on left ventricular function in the studied groups. (**a**) + dP/dt_max_, (**b**) left ventricular developed pressure, (**c**) end-diastolic pressure + dP/dt_max:_ maximum rate of increase in left ventricular pressure during systole, *IRI* ischemia–reperfusion injury, *Pre-IRI* Ischemic preconditioning + ischemia–reperfusion injury, *M-IRI* methadone + ischemia–reperfusion injury, *M-Pre-IRI* methadone + ischemic preconditioning + ischemia–reperfusion injury *P < 0.05, **P < 0.01 and, ***P < *0.001 vs*. Pre-IRI. **]** shows the comparison between Pre-IRI and M-Pre-IRI. *n* = 7 in each group.

+ dP/dtmax was the same in all groups at baseline. This index decreased significantly at the end of 60 min of reperfusion in all groups (*P* < 0.001). At the end of 60 min of reperfusion, dP/dtmax (contractility index) of the left ventricle of the Pre-IRI group was significantly higher than the IRI group (1831.9 ± 342 vs. 1254.9 ± 347.8 mmHg/s, *P* < 0.05). In the M-IRI and M-Pre-IRI groups, + dP/dtmax was similar to the IRI group and significantly lower than the Pre-IRI group (1158.5 ± 308.9 and 1143.4 ± 396.1 mmHg/s, respectively, *P* < 0.01) (Fig. 2a).

Left ventricular developed pressure was the same in all groups at baseline. However, at the end of 60 min of reperfusion, there was a significant decrease in all groups (*P* < 0.001). At the end of 60 min of reperfusion, the developed left ventricular pressure of the Pre-IRI group was significantly higher than the IRI group (53.9 ± 6.9 vs. 30.9 ± 8.3 mmHg, *P* < 0.001). In the M-IRI and M-Pre-IRI groups, it was similar to the IRI group and significantly lower than the Pre-IRI group (31.3 ± 6.9 and 30.8 ± 8.4 mmHg, respectively, *P* < 0.001) (Fig. 2b).

Left ventricular end-diastolic pressure was the same in all groups at baseline. However, at the end of 60 min of reperfusion, there was a significant increase in all groups (*P* < 0.001). The end-diastolic pressure in the Pre-IRI group was significantly lower than the IR group (28.4 ± 7.1 vs. 40.7 ± 7.4 mmHg, *P* < 0.05) and in the M-IRI and M-Pre-IR groups, it was similar to the IRI group and significantly higher than the Pre-IRI group (42.3 ± 6.5 and 43.1 ± 9.2 mmHg, respectively, *P* < 0.01) (Fig. 2c).

**Figure 3:**
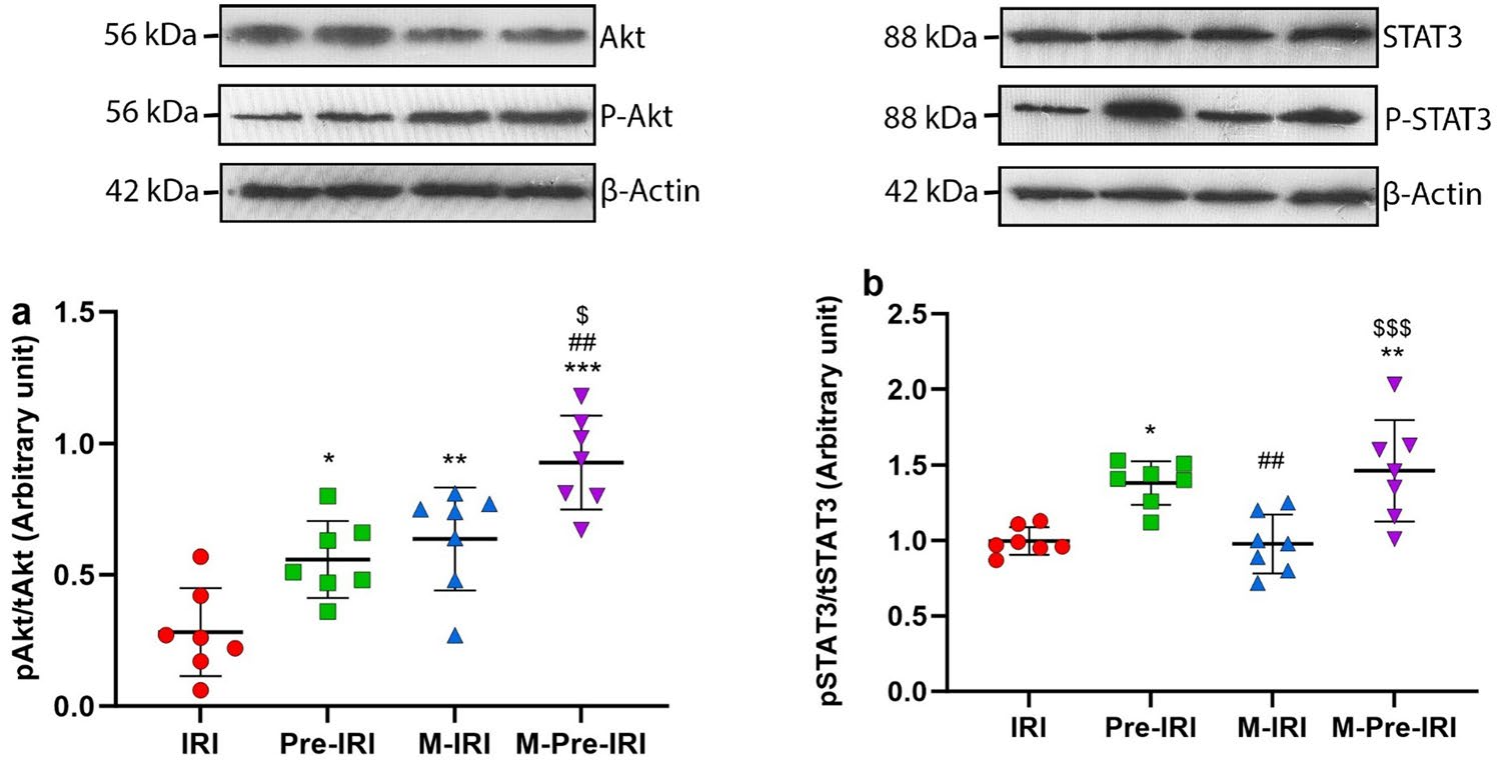
Effects of 4 weeks of methadone consumption on the pAkt/Akt (**a**) and pSTAT3/tSTAT3 (**b**) ratio in the studied groups. *IR* ischemia–reperfusion, *Pre-IRI* ischemic preconditioning + ischemia–reperfusion injury, *M-IRI* methadone + ischemia–reperfusion injury, *M-Pre-IRI* methadone + ischemic preconditioning + ischemia–reperfusion injury, *pAkt* phosphorylated Akt, *tAkt* total Akt, *pSTAT3* phosphorylated STAT3, *tSTAT3* total STAT3, *P < 0.05, **P < 0.01, ***P < 0.001 vs. IR, ^##^*P* < 0.01 vs. Pre-IR, ^$^*P* < 0.05, ^$$$^*P* < 0.001 vs. M-IR. *n* = 7 in each group.

The level of Akt phosphorylation in the Pre-IRI, M-IRI, and M-Pre-IRI groups was 0.56 ± 0.15, 0.63 ± 0.2, and 0.93 ± 0.18, respectively, which was significantly higher than the IRI group (0.28 ± 0.17) (*P* < 0.05, *P* < 0.01, and *P* < 0.001, respectively). In addition, the level of Akt phosphorylation in the M-Pre-IRI group was significantly higher than in the Pre-IRI and M-IRI groups (*P* < 0.01 and *P* < 0.05, respectively) (Fig. 3a and Supplementary File).

The level of STAT3 phosphorylation in the Pre-IRI group was 1.38 ± 0.14, which was significantly higher than the IRI group (*P* < 0.05, 0.99 ± 0.1), but in the M-IRI group (0.98 ± 0.2) it was similar to the IRI group. STAT3 phosphorylation in the M-Pre-IRI group (1.21 ± 0.3) was significantly higher than the IRI and M-IRI groups (*P* < 0.01 and *P* < 0.001, respectively) (Fig. 3b and Supplementary File).

## Discussion

The results of this study showed that chronic use of methadone for four weeks in rats had no effect against ischemia–reperfusion. In addition, chronic use of methadone inhibited the protective effects of preconditioning on ischemia–reperfusion injury in the heart.

During ischemic conditioning various factors such as adenosine, bradykinin, acetylcholine, opioids, TNF cytokines play a role. however, the opioid system is one of the most important systems in creating protective effects of ischemic preconditioning^33,34^. In addition, similar to ischemic preconditioning, acute administration of morphine five minutes before inducing ischemia–reperfusion injury reduces the infarct size and prevents arrhythmia after ischemia–reperfusion^12,13,15^.

However, in this study, we showed that chronic use of methadone not only did not have a protective effect against IRI but also blocked the protective effects caused by ischemic preconditioning. In line with our study, Cohen et al.^35^ observed in a study that in conscious ischemic rabbits, preconditioning, if applied for a short period, has a protective effect against ischemia–reperfusion, but if the ischemic preconditioning cycles are increased to about 40 to 65 cycles during 3 to 4 days before ischemia–reperfusion, it loses its protective effect, and if an interval without preconditioning occurs about 2.5 to 3 days before ischemia–reperfusion interval, a second period of ischemic preconditioning can exert a protective effect^35^. It appears that persistent ischemic preconditioning leads to persistent stimulation of the cardiac opioid system and exerts a similar effect to persistent stimulation of these opioid receptors by agonists. Long-term and continuous stimulation of the opioid system may cause tolerance to its beneficial effects on the heart. Tolerance to the analgesic effect of opioids is a known effect^36^.

Contrary to our study results, which showed that chronic administration of methadone had no protective effect against ischemia–reperfusion injury, Peart et al. showed that chronic use of morphine for 5 days made the heart of C57/BL6 rats more resistant to IRI and that these protective effects were exerted through DOR and KOR. In addition, the protection created in chronic conditions was about 20% higher than in acute administration, and even 48 h after withdrawal, the protective effect remained^37^. This difference can be due to the difference in the species of the studied animal, the duration of drug administration, the type of prescribed drug, and the type of receptor as methadone activates the mu receptor.

The opioid system is a fully integrated, coordinated, and complex system whose function is very precisely controlled, with many regulatory and compensatory mechanisms playing a role in its precise regulation^38^. This matter makes it highly adaptable. On the other hand, this feature makes it extremely vulnerable. For example, chronic exposure to morphine causes neurons to produce beta-endorphin 1–27, a weak morphine, instead of 1–31 beta-endorphin, which is a strong morphine. Therefore, in chronic use, not only are opioid receptors downregulated, but the system moves towards the production of weaker opioids^39^.

In this study, as expected, ischemic preconditioning increased the phosphorylation of Akt (activation of the RISK pathway) and STAT3 (activation of the SAFE pathway). These two pathways are the main protective pathways activated by ischemic preconditioning and improve heart function after IRI. Methadone alone increased Akt phosphorylation but did not affect STAT3 phosphorylation. It appears that these changes in the RISK and SAFE pathways inhibit methadone’s protective effect against IRI. In this regard, Goodman et al. showed in a study on male C57bl rats that in post-conditioning, the JAK-STAT signaling pathway is not able to create protective effects alone, without the activity of the PI3K-Akt pathway^40^.

Chronic use of methadone along with ischemic preconditioning caused a sharp increase in Akt phosphorylation, so the level of phosphorylation of this protein in the M-Pre-IR group was significantly higher than both the M-IR and Pre-IR groups. However, despite this sharp increase in activity in the RISK pathway, the protective effect of ischemic preconditioning was not observed. It has been shown that the continuous activation of Akt in the heart reduces cardiac resistance against myocardial infarction^41^. In addition, Akt hyperactivation inhibits the expression of antioxidant proteins through the inhibition of the forkhead family transcription factors^42^. Therefore, it appears that hyperactivation of Akt can be one of the reasons for the lack of protective effect of ischemic preconditioning.

Methadone reduces the size of the mitochondrial network, the number of mitochondrial objects, and increases the average area of mitochondrial objects. Also, methadone changes the shape of mitochondria. Thus, methadone can have a negative impact on mitochondrial structure, which may be linked to cell death^43^. Furthermore, in another study it was shown that methadone leads to necrotic-like cellular death by decreasing mitochondrial ATP synthesis, creating a bioenergetic crisis, and reducing the energy level of the cell^44^. Mitochondria play a crucial role in the cardioprotective mechanisms, particularly in the SAFE and RISK pathways^34^. Given the impact of methadone on mitochondrial function, it is plausible that chronic methadone administration can disrupt the cardioprotective mechanisms mediated by the SAFE and RISK pathways.

Research findings have demonstrated that Triiodothyronine and Gasterin can induce cardioprotection in isolated rat hearts by activating the RISK signaling pathway^45,46^. It would be intriguing to investigate whether activators of the RISK signaling pathway could have a cardioprotective effect in hearts with increased basal Akt activation due to chronic opioid receptor activation. This could potentially provide valuable insights into the mechanisms underlying cardioprotection in different physiological and pharmacological conditions. Further research in this area could help in understanding the complex interplay between different signaling pathways and their implications for heart health under various circumstances.

Ischemic conditioning, including preconditioning, postconditioning, and remote conditioning, have shown promising results in experimental studies for reducing ischemia–reperfusion injury in various organs, including the heart^5,47^. However, translating these strategies into clinical benefits for patients has been challenging^11^. One of the reasons for this challenge is the lack of understanding of the potential confounding factors that may impact the effectiveness of ischemic conditioning^48–51^. The present study highlights the potential role of chronic opioid receptor activation, specifically with methadone use, as a confounding factor that can inhibit the protective effects of preconditioning on ischemia–reperfusion injury in the heart. This suggests that chronic use of opioids, which is common in patients with chronic pain or opioid dependence, may interfere with the cardioprotective mechanisms of ischemic conditioning. In addition, our study helps to understand the mechanisms of ischemic preconditioning. Understanding its precise mechanisms has paved the way for the development of novel drugs and therapies that can mimic the effects of ischemic preconditioning and provide protection against ischemic injury,

A limitation of our study is the absence of a control group receiving a single dose of methadone for comparison with those using methadone chronically. Another limitation of our study is that we investigated a moderate conditioning stimulus, which may not have been sufficient to induce maximum cardioprotective effects. The translation of ischemic conditioning for cardioprotection in clinical practice has been challenging^50,52^. To address this issue, certain factors should be considered in pre-clinical studies^53^. For example, it has been shown that there is a dose–response relationship between stimulus potency and induced cardioprotection^54^. Thus, future studies should consider including different dosing regimens of methadone and a stronger conditioning stimulus to better understand the optimal stimulus strength for cardioprotective interventions.

## Conclusion

Chronic use of methadone eliminates the protective effects of preconditioning on IRI by changing the phosphorylation of Akt and STAT3 and possibly changing their signaling pathway. It appears that to observe the beneficial effects of cardiac preconditioning with opioids, special attention should be paid to the type of opioid and the duration of use. In addition, patients who use methadone and develop heart diseases may not be able to benefit from the beneficial effects of cardiac conditioning.

### Supplementary Information

Supplementary Information.

## Data Availability

The datasets used and/or analyzed during the current study are available from the corresponding author on reasonable request.

## Abbreviations

+ dP/dt _max_: Maximum rate of increase in left ventricular pressure during systole
Akt: Ak strain transforming
DOR: Delta opioid receptor
GPCR: G-protein-coupled receptor
IPC: Ischemic preconditioning
IRI: Ischemia–reperfusion injury
KOR: Kappa opioid receptor
LVDevP: Left ventricular developed pressure
LVEDP: Left ventricular end-diastolic pressure
M-IRI: Methadone + ischemia–reperfusion injury
MOR: Mu opioid receptor
M-Pre-IRI: Methadone + ischemic preconditioning + ischemia–reperfusion injury
pAkt: Phosphorylated Akt
Pre-IRI: Ischemic preconditioning + ischemia–reperfusion injury
pSTAT3: Phosphorylated STAT3
RISK: Reperfusion injury salvage kinase
SAFE: Survivor activating factor enhancement
STAT3: Signal transducer and activator of transcription 3
tAkt: Total Akt
tSTAT3: Total STAT3
OUD: Opioid use disorder
